# From food security to food wellbeing: examining food security through the lens of food wellbeing in Nepal’s rapidly changing agrarian landscape

**DOI:** 10.1007/s10460-016-9740-1

**Published:** 2016-10-18

**Authors:** Hom Gartaula, Kirit Patel, Derek Johnson, Rachana Devkota, Kamal Khadka, Pashupati Chaudhary

**Affiliations:** 1 International Development Studies, Menno Simons College, Canadian Mennonite University, 520 Portage Ave, Winnipeg, MB R3C 0G2 Canada; 2 Department of Anthropology, University of Manitoba, 432 Fletcher Argue Building, 15 Chancellor Circle, Winnipeg, MB R3T 2N2 Canada; 3 Local Initiatives for Biodiversity, Research and Development (LI-BIRD), Pokhara, Nepal; 4 Department of Plant Agriculture, University of Guelph, 50 Stone Road East, Guelph, ON N1G 2W1 Canada

**Keywords:** Food wellbeing, Agrarian change, Food security, Small-scale agriculture, Nepal

## Abstract

This paper argues that existing food security and food sovereignty approaches are inadequate to fully understand contradictory human development, nutrition, and productivity trends in Nepalese small-scale agriculture. In an attempt to bridge this gap, we developed a new food wellbeing approach that combines insights from food security, food sovereignty, and social wellbeing perspectives. We used the approach to frame 65 semi-structured interviews in a cluster of villages in Kaski district in the mid-hills of Nepal on various aspects of food security, agriculture, off-farm livelihood opportunities, and women’s wellbeing. Our results indicate that context-specific subjective and social relational factors highlighted by the food wellbeing approach are key to understanding a paradox of increased food security, yet decreasing sustainability of small-scale agriculture. Increased levels of male out-migration and opportunities for local off-farm work have increased local capacity to purchase food. The positive consequences for food security are indicated by evidence that households with non-farm income sources had better food sufficiency, absorption capacity, nutritional quality, and stability of food supply. These off-farm employment opportunities have also led to the greater involvement of low caste groups and women in small-scale agriculture. This has been empowering for both groups and led to an increase in wellbeing, particularly for those women who have become de facto heads of household. Yet, small landholdings, persistent patterns of unequal and absentee land ownership, sharecropping, women’s overwork, and the aspirations of low caste farmers and women away from agriculture are simultaneously driving the erosion of local small-scale agricultural productivity and ecological sustainability.

## Introduction

In the past decade, Nepal has made remarkable progress towards achieving food and nutrition security. In the period between 1990 and 2013, the percentage of the population living below one dollar a day has decreased from 34 to 16 %; the prevalence of underweight children below five years of age has declined from 57 to 29 %; and the prevalence of stunting in children below five has declined from 57 to 41 % (GON/UNDP 2013). Indicators of life expectancy (69 years), child mortality (50 children U5/1000 live births), maternal mortality (170 children U5/100,000 live births) and adult literacy (60 %) are better than or comparable to levels in many developing countries (UNDP 2013). At the same time, however, farm production and productivity are declining. This is in part due to an agricultural system vulnerable to climate change, especially through an increasingly uncertain monsoon, the effects of which are further worsened by the country’s complex and diverse terrain (Chhetri et al. 2012). Studies indicate that Nepal is becoming food insecure due to environmental, social, cultural, political and economic factors (Gaire et al. 2014; Chapagain and Gentle 2015). In 2012–2013, Nepal imported over US$200 million worth of cereals and almost US$50 million worth of vegetables from other countries (GON/UNDP 2013), signs of decreasing food self-sufficiency in Nepal.

Nepal has gone through rapid political changes in the past three decades, which have been accompanied by increased access to information about persistent inequities in the country. The establishment of democracy^1^ has opened up opportunities for development, but rising expectations have not been adequately fulfilled. Democratization has been coupled with another set of changes: the deregulation of agricultural policies that included the lifting of subsidies on agricultural inputs, which was influenced by the structural adjustment programs of the International Monetary Fund (IMF) and the World Bank to promote growth through economic liberalization. These changes have had an impact on the people of Nepal at different levels and scales, making small and marginal farmers more vulnerable, as research shows that these programs have had a negative impact on poverty levels and income distribution (Oberdabernig 2010). The agricultural sector—accounting for over two-thirds of the country’s labour force and one-third of national gross domestic products—has become a less attractive source of occupation for rural youth (Gartaula et al. 2012a). Consequently, rural Nepalese have looked for opportunities outside agriculture and frequently even outside the country (Lecomte-Tilouine 2009). This has changed collective perceptions about earning a living and accessing food in Nepal.

The increasing trend of Nepali youth going abroad to work in the Gulf or in other Asian countries^2^ is indicative of Nepal’s changing agrarian labour landscape. Over 94 % of these migrant workers are male youth (DOFE 2013) who leave their female counterparts to stay behind and manage agriculture alongside their traditional domestic chores. Though women have always been an important part of the agricultural labour force in Nepal,^3^ the current agrarian transition has changed women’s position from mere agricultural co-workers to de facto household decision-makers. When men take up more non-agricultural activities, women’s responsibilities for agriculture and household management increase (Adhikari and Hobley 2015; Zuo 2004; Radel et al. 2012; Kelkar 2009). This can be experienced as a burden or overload, but also as an opportunity for increased agency, empowerment and capacity building (Kaspar 2006; Gartaula et al. 2010). These subjective perceptions of rural Nepalese women are shaped in response to, and feed into, the local and external changes that are taking place, and thus must be considered by development scholars and policy analysts.

Several recent studies (Sharma 2008; Chhetri et al. 2012; Gaire et al. 2014; Chapagain and Gentle 2015; Adhikari and Hobley 2015) highlight the intricate challenges Nepal faces in achieving food and nutrition security. These studies focus on scale, causes, agroecological challenges related to food production, technological interventions and institutional or policy initiatives for addressing food and nutrition insecurity. However, they fail to take into account the subjective and social relational experiences of women and low-caste groups who make household-level livelihood choices around food security and wellbeing.

Considering the increased role of these two groups in agriculture, the future of family farms and long-term food security are directly related to their subjective and relational experiences as they navigate livelihood processes, outcomes and impacts. Our guiding research question is, thus, how do changing agrarian and labour landscapes shape food security, livelihood choices and the wellbeing of those who continue to engage in local small-scale agriculture? Using the approach we have labelled food wellbeing, this study aims to understand the interactions among household livelihoods, food security and the wellbeing of left-behind women and lower-caste farmers. The term *food wellbeing* synthesizes key insights from food security, food sovereignty and social wellbeing approaches to generate a better understanding of the complex nature of food security in the context of current agrarian change, and the subjective perceptions of women about those changes.

## Literature review and conceptual framework

### Food security: definition and approaches

Food security is a vital component of human development and wellbeing that must be safeguarded and sustained by states, communities and individuals. The concept of food security was first introduced to the global community at the World Food Conference organized by the United Nations General Assembly in 1974. Since then, there has been considerable debate among researchers and policymakers about the conceptualization and measurement of food security. The emergence of over 200 definitions and 450 measurement indicators of food security is evidence of this (Mechlem 2004; Maxwell 1996). The 1970s definition of food security was influenced by fluctuations in food supply due to production constraints and instability of food grain prices (FAO 1974). As time went on, the unequal access to and distribution of food—due to a lack of economic resources and individual capabilities—were argued to be equally important aspects of food security. This led to a distinction between the ability of the state to ensure a constant supply of food at the national level, and the capability of individuals or households to access available food and subsequently contributed to FAO’s widely accepted multi-dimensional definition of food security (Sen 1981; Drèze and Sen 1989; Watts and Bohle 1993; FAO 1996).

Yaro (2004) categorises approaches to food security into three major groups: the *food availability* approach; the *livelihood and entitlement* approach; and the *food sovereignty* approach. According to the food availability approach, the primary cause of food insecurity is a lack of food, and thus it emphasizes an increase in the production and storage of food grains at regional and national levels (Maxwell and Frankenberger 1992). The food entitlement and livelihood approach is based on the premise that hunger and malnutrition are caused not only by inadequate food supply but also by a lack of purchasing power on the part of the poor to meet their food requirements (Sen 1981). If people gain access to income or the means to earn a livelihood, they can purchase food from the market, which reduces the direct production of food as a necessary condition for food security (Osmani 1993). In the entitlement approach—also considered as a subset of the livelihood approach—food security is viewed as an integral dimension of livelihood security, which is shaped by a household’s access to a diverse set of endowments as well as by its capabilities to convert these endowments into entitlements and services (Scoones 1998). The food sovereignty approach, which is drawn from a human rights perspective on poverty, hunger and malnutrition, gives farmers a central role in defining their own food and agriculture system, and in protecting and regulating agricultural production and trade to achieve self-sufficiency and sustainable development, the keys to food security (Patel 2009). According to this approach, food security is attained when small farmers have access to land and the sovereign right to select, cultivate, consume, exchange and trade their own crops (Pimbert 2009; Altieri 2009). It advocates local small-scale farmer decision-making autonomy in order to promote ecological sustainability and the preservation of nutritional culture through diversity of cultivated food crops (Menezes 2001). In other words, the food sovereignty model seeks to strengthen the agency of small family farms and the peasantry to reorganize existing systems of food production controlled by agricultural input providers and the food processing industry (Holt-Giménez and Shattuck 2011). This approach emphasizes the use of agroecological principles in farming, local markets and consumption, and gender equality to achieve national food sufficiency through small-scale family farms.

The focus on family farms under the food sovereignty approach is not without its critics, however. Agarwal (2014) argues that the issue of gender inequality in agrarian landscapes is complex, and difficult to address through the family farm, where women experience significant marginalization. The problems faced by women farmers are structural and deep-rooted and cannot be solved simply by increasing women’s space for agency and decision-making (Rao 2006). The family farm is often a less attractive livelihood option for women unless productive assets such as land and agricultural inputs are redistributed in gender-equal ways, and the state reorients its agricultural research and extension services to address technological constraints faced by women farmers. These issues reinforce the importance of examining the subjective experiences of rural women in their roles as farm co-workers, decision-makers and de facto heads of the household in order to fully understand the impacts of various food security approaches.

The three major approaches for addressing food security differ in their strategic foci, ranging from the *means* of attaining food security to the *ends*, or outcome, of being food secure (Patel et al. 2015), but all of them emphasize four pillars of food security: availability, access, utilization and stability (FAO 2006). Food availability refers to the disposition of sufficient food in appropriate quality, which can be supplied through domestic production, imports through markets or by food aid. It is the physical availability of food in a country or region by any means, while food access refers to household or individual ability to obtain food by means of economic security. This dimension emphasizes economic capability, legal or traditional rights (entitle-ments), and political and social arrangements of populations to access food for their dietary requirements. Food utilization focuses on the nutritional requirements for and absorptive capacity of the human body. Access to and adequacy of dietary resources, clean water, sanitation and health care are the essential conditions for this pillar to assure the nutritional wellbeing of an individual, which thereby points to the importance of non-food inputs to food security. Finally, the stability dimension calls for a regular and assured supply of food, with minimal risks in situations of economic and climatic crisis (shocks) or seasonality (cyclical events). Thus, the pillar of food stability depends on both availability of and access to food (FAO 2006).

### From food security to food wellbeing: a conceptual framework

The above review of current food security literature reveals that its main emphasis is on physical and economic access to food and the biological and bodily utilization of food, rather than on the social and cultural factors that shape food preferences and access to food (Noack and Pouw 2015; Craven and Gartaula 2015). People do not simply strive to increase their physical supply of food, as the food availability approach suggests. Neither do they only seek to build their capability to access food, as the entitlement and livelihood approach believes, or simply seek the freedom to make food-related choices, as the food sovereignty approach suggests. People also persistently look for ways to improve their wellbeing in ways that are meaningful to them.

In order to bring these nuances to food security scholarship, this paper draws upon the concept of social wellbeing, which is defined as “a state of being with others, which arises where human needs are met, where one can act meaningfully to pursue one’s goals, and where one can enjoy a satisfactory quality of life” (McGregor 2008, p. 4). Social wellbeing embraces a three-dimensional approach to assess human wellbeing outcomes: objective, or material; subjective, or cognitive; and relational (Gartaula et al. 2012b). “The material dimension emphasizes the resources people have and the extent to which the needs of the person are met; a relational dimension which considers the extent to which social relationships enable the person to act meaningfully in pursuit of what they regard as wellbeing; and a cognitive dimension which takes account of their level of satisfaction with the quality of life they achieve” (Britton and Coulthard 2013, p. 29). In the context of food security studies, these material dimensions relate to the availability of food, access to food, ability to use and make effective choices with regard to food, and subjective perceptions about the overall quality of food and the immediate as well as long-term impacts of household livelihood processes. These considerations relate to people’s interactions with others, their agency, and relations with the state, social institutions, rules and norms, which can dictate access to food (Britton and Coulthard 2013; Craven and Gartaula 2015; Noack and Pouw 2015; McGregor 2006). This integration of the three-dimensional wellbeing perspective with insights from the food availability, food entitlement and livelihood and food sovereignty approaches is what we call the *food wellbeing* approach. Food wellbeing is thus a state where people are able to produce, choose, and consume food that is socially, culturally, ecologically appropriate and calorically, nutritionally, and subjectively satisfying.

**Fig. 1:**
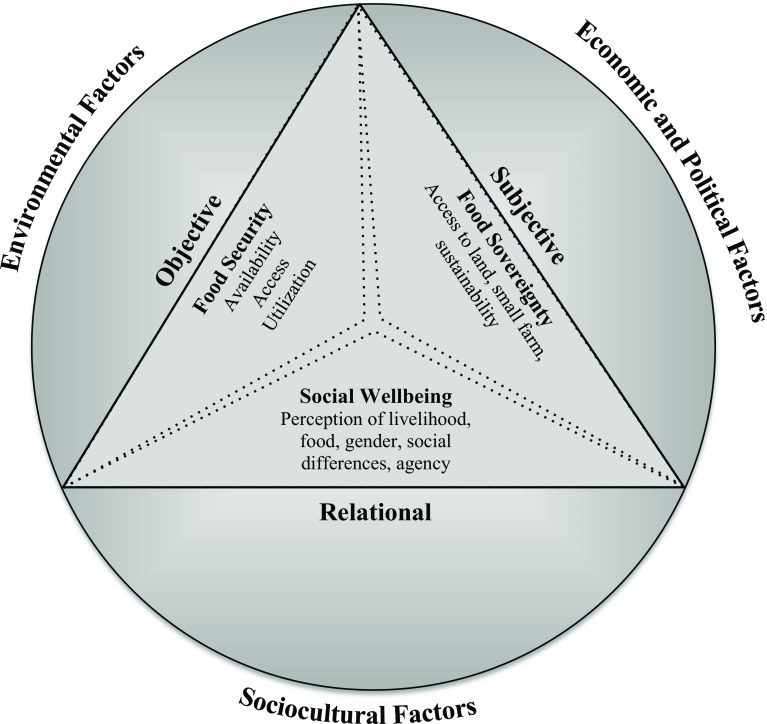
Conceptual framework of the food wellbeing approach

A schematic diagram of the food wellbeing approach employed in this study is depicted in Fig. 1. The outer circle represents the food wellbeing of an individual or a household, which has a two-way relationship with social, economic, political, cultural and environmental factors. The inner three sub-triangles pointing towards the centre indicate that we need to consider food security, food sovereignty, and social wellbeing when assessing attainment of food wellbeing. The dotted lines of three sub-triangles show that we do not wish to compartmentalize these three approaches, but to highlight complementarities and intersections between them. We do not intend to suggest that there is an exclusive association between each theoretical approach and the particular aspect of wellbeing on each side of the triangle even if we have chosen to explain the approaches primarily on the basis of these associations. The *objective* aspect of the proposed food wellbeing framework in our research relates to food availability, access, sufficiency, quality and capacity to utilize, areas that are of particular interest to the food security approach. The *subjective* aspect speaks to how land distribution, sharecropping mechanisms, ethnicity and socioeconomic inequalities (all relational factors) influence *perceptions* of agricultural livelihoods and thus shape long-term food production and the sustainability of small-scale agriculture. As the food sovereignty approach is deeply interested in these issues, we have placed it adjacent to the subjective dimension, although clearly these issues are also of direct relevance to social wellbeing. The latter approach draws attention to other *relational* concerns connected to accessing food, with particular attention to gender and the influence of other social differences on decision-making, and the agency of local actors as they make immediate and future food choices for their food security.

Consistent with the holistic and relational logic of the social wellbeing perspective, the food wellbeing approach ingrained in our research provides an integrated framework for understanding food security that more fully brings out subjective and social relational factors. This increased attention to subjective and relational dimensions deepens the possibility of understanding the opportunities and contradictions of agrarian change. In our focus case, it shows how overemphasis on objective measures of food security may pose the risk of concealing the simultaneous erosion of food sovereignty and potentially, even, the threat of long-term food insecurity. Simply put, it provides a space for consideration of subjective and relational factors, such as access to land and other productive resources, empowerment and agency, gender and social differences, and aspiration for a better future.

## Methods

### Research location and site selection

The research for this paper was conducted in one of the Village Development Committees (VDCs) of Kaski district, in the Western Development Region of Nepal. Located about 200 km west of the capital city of Kathmandu, Kaski is a relatively affluent district among the 75 districts of Nepal. The research VDC has a total population of 7318 (55 % women) living in 1880 households. The literacy rate of the VDC is 64 % (Karthikeyan et al. 2012), which is much lower than the district average of 83.9 % (91.5 % for men and 77.0 % for women) (CBS 2011a). The research site is located between 841 and 2074 m above sea level, which gives it considerable diversity in topography, climate and vegetation across the slope. The lower elevation has a warm, humid and sub-tropical climate, while the higher elevation harbours a cold, dry and temperate climate, resulting in two distinct cropping patterns: a rice-based pattern in the warmer, lowland riverbank areas, and a maize-millet–based pattern in the colder, upland areas. Agriculture is largely subsistence-oriented and is practised by small and marginal farmers.^4^

The research site was one of the eight project sites in South Asia for a large interdisciplinary research project on food and nutrition security through the promotion of small millets conducted by a consortium that included partners from academic and research institutions in South Asia and Canada. In Nepal, the project was coordinated by an NGO called Local Initiatives for Biodiversity, Research and Development (LI-BIRD). Our field research was conducted in the four selected villages of the research VDC, distributed in the four wards. These villages were selected in consultation with the LI-BIRD staff and key informants, based on the prevalence of diversified livelihood portfolios, including rural–urban migration, small-scale agriculture and local off-farm activities.

### Research design, data collection and analysis

A mixed methods research design that combines quantitative and qualitative methods of data collection was required for this study to be able to address the holistic data needs of the three conceptual approaches (food security, food sovereignty, and social wellbeing) that together constitute the food wellbeing approach that we apply to analyse the effects of agrarian change in Nepal. In particular, we required both quantitative and qualitative data to fully capture the objective, relational and subjective aspects of food wellbeing. The quantitative data were collected using a semi-structured survey, while qualitative methods included participant observation, focus group discussions and key informant and in-depth interviews. The fieldwork was conducted in 2012 and 2014, and was supported by two enumerators who conducted the 2012 survey. To avoid repetition and save time, a household list, which LI-BIRD had used for a baseline survey of their project, was made available and used for this study to prepare a sampling frame. The list was verified with the help of village elders, and from the final list of 717 households, one-ninth of them were selected for interviews using systematic random sampling. This created an initial sample of 79 households. Nine households, however, could not be found during home visit attempts or did not respond, leaving a sample total of 70 households. The survey asked questions about the socioeconomic and demographic profiles of the research participants, including information about landholding, agriculture, food self-sufficiency and objective and subjective measurements of wellbeing. An additional 68 individuals were consulted through eight focus group discussions, 24 individual interviews were conducted and contextual data was gathered through the researchers’ observations and informal interactions with research participants.

As per the framework described earlier, we incorporated all three aspects of wellbeing into a wellbeing index of participants that measured several variables related to the level of access to resources (income, housing and drinking water), as well as the adequacy of resources (income, housing, drinking water, child education, health care facilities, and emotional and social relationships). Emotional relationships referred to the relationships among household members, while social relationships were defined as the relationships of households with other community members. The index also included a subjective evaluation of the participants’ quality of life over the past 12 months, based on overall life satisfaction and perception of their quality of life in relation to that of other members in the community. These 12 variables were measured on a three-point scale with the following grading: (1) low; (2) medium; and (3) high accessibility, adequacy or satisfaction, as experienced by the respondent. The individual responses for these variables were combined to create the participants’ overall wellbeing index, with scores ranging from 12 to 36.

The majority of respondents were from Brahmin-Chhetri^5^ groups, followed by respondents from Dalit^6^ and Janajati^7^ groups. As livelihood choices and people’s wellbeing largely depend on landholding, which, among other factors, is based on caste and ethnic identity, the households were categorised based on caste/ethnicity. In order to develop a more explicit analysis of agriculture plus engagement of households within and outside their village, five households were deliberately taken out of the sample group, as they did not have agricultural land. The remaining 65 households were first categorized according to their engagement in local and distant non-agricultural activities. The households with local engagements were further grouped into informal and formal sector involvement, whereas households with distant engagements were categorised based on the relationship of the person involved to the household head. This classification resulted in four household categories (Table 1): HHAI (households primarily based on *agriculture* and the *informal* sector (typically agricultural wage labour) within the village); HHAF (households based on *agriculture* and the *formal* sector within the village); HHDH (households where the *head* of the household was involved in *distant* off-farm activities); and HHOD (households where *other* members of the household were involved in *distant* off-farm activities).

**Table 1:** Household categories by occupation, household structure and caste

Household categories (N = 65)	Total (n)	%
*By occupation*		
Agriculture and local off-farm work		
Informal sector (HHAI)	18	27.7
Formal sector (HHAF)	10	15.4
Agriculture and distant off-farm work		
Household head involved (HHDH)	22	33.8
Other members involved (HHOD)	15	23.1

	Nuclear (%)	Joint (%)	Total (n)	%
*By household structure (N* *=* *65)*				
Male-headed	27	73	35	53.8
De facto female-headed	100	0	22	33.8
De jure female-headed	37	63	8	12.3

	Total (n)	%
*By caste/ethnicity (N* *=* *65)*		
Brahmin-Chhetri	39	60.0
Janajati	5	7.7
Dalit	21	32.3

For in-depth understanding of the gender dynamics in the area, the households were also categorized based on household headship. According to whom respondents considered to be the head of the household, 88 % were male-headed and 12 % were female-headed households. However, when men enter into non-agricultural jobs, especially outside the village, women often run their households as acting heads. Out of all households sampled, 33.8 % were de facto female-headed. In these households, men are considered the head, but in their absence women take on an acting role of household head. All de facto female-headed households were nuclear households and women were responsible for day-to-day decision-making. We thus came up with three categories of households: male-headed, de facto female-headed, and de jure female-headed. In terms of household structure, 27 % of male-headed, 100 % of de facto female headed and of 37 % de jure female-headed households were nuclear in composition (Table 1). The increasing number of de facto female-headed households is an important consideration in the Nepalese sociocultural context, as it alters gender norms and intra-household power relations. Considering the importance of women’s perceptions of their wellbeing, we decided to exclude six male respondents from the sample while pursuing in-depth quantitative analysis of women’s wellbeing. This resulted in a sample size of 59, instead of 65, for the statistics presented in Table 4 on women’s wellbeing.

The qualitative data were analysed through qualitative content analysis, which allows researchers to understand social reality in a subjective but structured manner by examining the meanings, themes and patterns that manifest in particular texts (Zhang and Wildemuth 2009). Excel and IBM Statistics 19 were used to complete descriptive statistics, correlation and ANOVA from the quantitative data.

## Results and discussion

He was already a migrant worker at the time of our marriage. I continued farming for some time after marriage, but despite working hard in our fields, we didn’t get a good return. We can produce grains, but our life needs more than just food. How can we pay school fees, how can we buy clothes, how can we pay for medicine? Let alone saving for our children’s marriages, adding property or buying jewellery! I discussed this matter with my husband and I started this shop five years ago by investing money he made abroad. There is not a big profit in the shop, yet it is far better than working on the farm. It is easy and also gives cash income. […] We have to make a living in any way possible. Production from agriculture is not enough. What would he do if he were here? It is difficult to make money staying here; they [husbands] have to go out. The money earned abroad is visible, but money earned locally is spent without notice. […] I don’t want his income to be spent here and there. His income is for saving for big things like buying land or building a house. Daily household expenditures, school fees and smaller health costs are covered from the income I make from this shop. We do not have to buy food grains, as we have rented our land out for sharecropping and our share is enough for food.

This interview transcript from a de facto female household head, whose husband has been working abroad for over 14 years, helps illustrate the context of our research. It sheds light on perceptions of livelihood and food security, the drivers motivating local men to seek off-farm livelihood activities outside of the village, and the types of household projects designated for remittance money. It also shows women’s increasing role in household decision-making, the maintenance of household food security, and women’s perceptions of food, land and agriculture as resources for balancing food security and wellbeing. These factors collectively point to food security as more than just secure access to food. While this case features a household with a member who has out-migrated, the attitude towards agriculture and perceptions of food security among non-migrant households are similar. Starting with the description of respondent households, the remaining empirical sections revolve around the experience of respondents differentiated by class, caste, gender and other social inequalities. These axes of social difference shape access to land and other resources needed to engage in different livelihood activities within and outside their village. Our data also highlight the struggle of villagers to find a balance between food security and wellbeing in the pursuit of a livelihood in a changing agrarian landscape.

### Profile of the respondent households

The majority of respondents were middle-aged (average age 43.5 years), female (90.8 %) and married (86.2 %). Almost half were literate, with an average of eight years of school education. Sixty-nine percent of respondents were the spouse of the household head. About 94 % were Hindu and the rest followed Buddhism as their religion. The average monthly income was NPR 12,506 (US$125). The majority of respondents (71 %) lived in semi-pucca houses, while 17 % lived in pucca houses and the remaining 12 % in kachcha houses.^8^ Over 90 % had access to drinking water provided through public water taps and private pipelines connected to their houses; the rest had to fetch water for drinking from open wells and springs.

Even though agriculture remains the most common livelihood activity for respondent households, non-agricultural occupations are increasingly important in the research area. The overwhelming majority of households (94 %) had income from more than one occupation, and only 58.5 % reported agriculture as the main occupation. Other occupations included foreign employment (20 %) and government jobs (15.4 %). The rest (6.1 %) were engaged in wage labour, private sector jobs and small businesses.

### Food self-sufficiency: availability and access

**Fig. 2:**
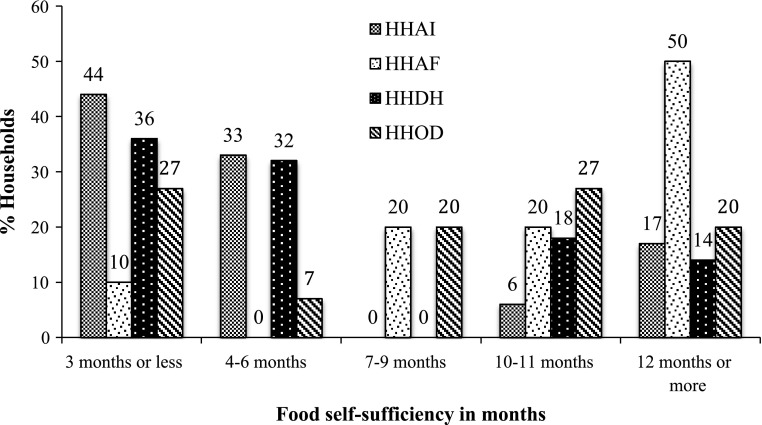
Food available from the stock of self-production. *HHAI* Households based on agriculture and local informal sector off-farm income; *HHAF* Households based on agriculture and local formal sector off-farm income; *HHDH* Households based on agriculture and distant off-farm income, household head involved; *HHOD* Household based on agriculture and distant off-farm income, other household members involved

Results show that all household categories, as we defined them above, have a limited food supply from their own production for year-round consumption. This limit on self-produced food is compensated for by access to income from non-agricultural off-farm activities, both within and outside of the village. In other words, there is no food insecurity in the area. Only one surveyed household had skipped a meal in the previous 12 months. Over 78 % of households purchased food using non-agricultural income from both distant and local work. While distant non-agricultural income included remittances, local off-farm income sources included wage labour, pensions and profits from local businesses, and salaries from employment in the formal sector, which in most cases is teaching in the local schools. Figure 2 shows that of all household categories, only HHAFs have a majority of households with access to self-produced food for more than six months in a year. Even though they rent out most of their cultivated land (see Table 3 in the next section for landholding among household categories), HHAFs have the highest level of food self-sufficiency because they have the largest landholdings and are supported by secure incomes from formal sector jobs within the village.

As food entitlement and livelihood approaches suggest, food can be obtained not only from self-production but also through the economic capacity of people to access food markets. Our data show that households dependent on agriculture and local informal wage labour (HHAI) have the lowest household income (NPR 9000/month), which is understandable, as agriculture is practised mainly for subsistence and rural wages from other sources are low. In contrast, households that are involved in agriculture and formal sector off-farm activities within the village (HHAF) have the highest incomes of all household types (NPR 17,000/month). The households involved in distant off-farm activities (HHDH and HHOD) fall between the first two household types in terms of monthly household income. The data show that food availability and access are not a big issue in the research area. However, it is important to note that for a landlocked country like Nepal, food self-sufficiency from domestic agricultural production is crucial for a sustainable solution to food security, as economic security may not always suffice as a means to obtain food, as the recent land blockade of the Indian border showed.^9^

### Quality and absorption of food: crop diversity, livestock, housing and sanitation

Food security is more than just the physical availability of and economic access to food. Food must be nutritionally diverse and should exist in a form that healthy bodies can effectively use or absorb. Safe drinking water, housing and sanitation quality, and local availability of diverse food crops and animal products are important for better absorption and dietary diversity. Apart from mainstream crops like rice, wheat and maize, the farmers also cultivate nutritionally rich crops such as finger millet, black gram, soybeans, other beans and green vegetables in their homestead gardens and on other suitable land, depending upon their social, economic and cultural preferences. These crops are not only the primary sources of dietary fibre, proteins, vitamins and minerals; they also enhance dietary diversity in everyday meals.

In addition to crop diversity, the integration of livestock is another salient feature of small-scale agriculture prevalent in the area. Crop-livestock integration plays an important role not only in ecological sustainability but also in providing an additional supply of proteins, vitamins and minerals to the household diet (Altieri 2009). Respondents were found to have large (cattle, buffalo) and small (goat, sheep) ruminants and poultry in their homesteads, mainly for ploughing and producing manure, milk and meat. The statistics presented in Table 2 show that households that have members engaged in distant off-farm activities (HHOD and HHDH) have high crop and livestock count compared to households engaged in the local informal sector (HHAI). Interestingly, households with employment in the formal sector (HHAF) continue to keep more livestock despite their tendency to rent out their land. In-depth analysis of quantitative indicators of crop diversity [r(65) = 0.29, *p* < 0.05] and livestock count [r(65) = 0.41, *p* < 0.01] indicated significant positive correlation with the household size. The HHAI households not only had smaller size, but also most of their family members were engaged in local wage labour and were not available for livestock care and agriculture. This affected their access to crop diversity and to livestock. Recent studies indicate that farm diversity (including crop and livestock diversity) is positively correlated with household dietary diversity (Jones et al. 2014; KC et al. 2015). Sthapit et al. (2008) observe that farm diversity is a crucial asset available to farmers for managing vulnerability, uncertainty, shocks and stresses against climate change. They also note that sociocultural factors shape the preferences and tastes of food prepared from specific crops and crop varieties, indicating a significance of subjective experience for food wellbeing.

**Table 2:** Crop diversity and livestock population as indicators of quality and nutritious food (N = 65)

Household type	N	Crop diversity	Crop and livestock count	Household size
Agriculture and local off-farm				
Informal sector (HHAI)	18	2.5	5.4^a^	4.6^a^
Formal sector (HHAF)	10	2.4	6.4	4.5^b^
Agriculture and distant off-farm				
Household head (HHDH)	22	3.1	6.2	5.0
Other members (HHOD)	17	2.5	8.1^a^	5.7^ab^

^a,b^Indicate that statistical means with the same letter in the same column are significantly different at 0.1 level (one-way ANOVA)

We also examined the capacity of household members to utilize available food based on their access to housing, sanitation and the source of drinking water. Altogether 44 % of HHAIs live in kachcha houses, whereas none of the other household types live in this type of housing. While none of HHAIs live in pucca houses, 30 % of HHAFs, 23 % of HHDHs and 20 % of HHODs live in superior pucca houses. All other respondents live in semi-pucca houses. Similarly, 44 % of HHAIs have pit latrines while none of the other household types have to rely on pit latrines. Impressively, 20 % of HHAFs, 5 % of HHDHs and 13 % of HHODs have automatically flushing modern toilets, while all others have modern toilets with manual flushes. The area is connected to the public water supply, but 33 % of HHAIs still depend on natural springs for drinking water. Some HHAFs, HHDHs and HHODs have connected their homesteads through private pipes to the public water taps. These figures show that households engaged in off-farm economic activities have better access to higher quality housing and sanitation, and are thus likely have greater capacity to absorb nutrients from the food they eat.

### Access to land and sharecropping: examining food sovereignty

The research area is characterized by small-scale agriculture with average landholdings of 1.18 acres per household (Table 3). The distribution of landholding in Nepal is not only a matter of economic capability but also a sociocultural matter in which the caste system and ethnic identity are important, reflecting the history and geopolitics of the country. Upreti (2008) reports that after the geographic unification of Nepal by Shah (Chhetri) rulers, beginning in the eighteenth century, Brahmins-Chhetris consistently occupied the core of the state machinery, which increased their access to land and other productive resources. The HHAF category, which largely comprises Brahmin-Chhetri households (80 %) and includes no Dalit households, has the highest average land ownership (2.05 acres), compared to other household categories that have a strong presence of Dalits (39 % in HHAI, 36 % in HHDH and 40 % in HHOD categories).

**Table 3:** Landholdings and household income as indicators of food availability and access

Household categories	Landholdings (acre)			
	Owned	Cultivated	Rented in	Rented out
Agriculture and local off-farm				
Informal sector (HHAI)	0.89 (18)^a^	0.92 (18)^a^	1.07 (6)	1.18 (5)
Formal sector (HHAF)	2.05 (10)^ab^	1.26 (10)	0	1.57 (5)
Agriculture and distant off-farm				
Household head (HHDH)	0.80 (22)^bc^	1.04 (22)	1.58 (5)	1.24 (2)
Other members (HHOD)	1.54 (17)^c^	1.69 (17)^a^	2.28 (4)	1.71 (4)
	1.18 (65)	1.19 (65)	1.56 (15)	1.44 (16)

Figures in parentheses indicate frequencies. ^a,b,c^ Indicate that statistical means with the same letter in the same column are significantly different at 0.05 level (one-way ANOVA)

Landholding has generated three patterns of labour arrangement in the research area: those who cultivate their own land; those who cultivate others’ land through sharecropping or lease arrangements; and those who have land but lease it out for sharecropping or fixed-term contracts. Sharecropping is linked to ethnic distribution in the village, although its trend, pattern and scale are changing. Traditionally, hill villages were a patchwork of ethnic or caste-specific settlement areas. In the research VDC, Brahmin-Chhetris are the dominant groups with patches of settlements. In many parts of the VDC, Janajatis and Dalits have their own settlement clusters. For example, Gurungs (a Janajati group) are the primary inhabitants of Ward 6. Ward 4 is inhabited by Dalits (mainly of the blacksmith caste). Dalits were found to have smaller average landholdings (0.52 acres) than Brahmin-Chhetri (1.37 acres) and Janajati (2.56 acres) groups. None of HHAFs (agriculture with formal employment) rent land from other owners, while half of them rent their land out. Participants’ stated reason for renting land was not having enough agricultural land of their own to cultivate, while the reasons for renting out their land were children not wanting to work on the farm, household members showing no interest in agricultural work and household members engaging in off-farm activities, thus resulting in an unavailability of household labour to work on the farm. Among the 18 HHAIs that largely depend on agriculture, 33.3 % rent land while 27.7 % rent out land.

Dalits are the occupational castes that traditionally worked to make farm tools, domestic utensils, clothing, shoes, etc., and in return they would receive food grains during harvest season. Now many of these households do sharecropping, as mass-produced goods have undermined their traditional economic activities (Mills 1997). The craft skills of Dalits have potential conversion value into industrial manufacturing, as has occurred to some degree in countries like Bangladesh, but this has not occurred in Nepal. Consequently, the culture of a reciprocal local economy based on patron-client relationships between upper and lower caste groups (Darnal 2011) has been undermined, forcing local artisans to look for alternative livelihood options within and outside their villages. Dalits have therefore sought to expand their livelihood portfolios through sharecropping or foreign labour employment, since local service sector employment has not been easily accessible to them. Among the 21 Dalit households, 52 % rent land from others, while only one household rents their land out, and 66.7 % have members working in the foreign labour market. In contrast, out of 39 Brahmin-Chhetri households, 31 % rent out their land. Renting land from others seems to have provided better access to diverse and thus nutritious food for Dalits, as data show a positive correlation between crop diversity and land renting-in behaviour [r(63) = 0.29, *p* < 0.05], while this relationship is negative with land renting-out behaviour [r(63) = −0.46, *p* < 0.01]. It is also evident that Dalits have higher diversity of crops and livestock, which reinforces the likelihood of their greater food wellbeing compared to other caste groups. An empirical study (Karthikeyan et al. 2012) carried out in the same VDC by the RESMISA project found that the Dalits had a higher proportion (50 %) of children (below 6 years) with normal or healthy body mass index (BMI) than other castes such as Brahmin-Chhetri (36 %) and Janajati (33 %).

In terms of labour organization, Dalits appear to have moved from wage labour to sharecropping, which can be seen as a better employment opportunity than casual labour, especially in terms of agency and access to resources. From this perspective, it can be argued that Dalits have increased their access to land resources through sharecropping and thus have become more empowered. De Janvry and Sadoulet (2001) note that sharecropping may be a pathway to land ownership. In fact, Adhikari and Hobley (2011) found an increasing tendency of Dalit households to buy land in the village using remittance money. In the case of Dalit households, the agrarian transition in the research area may be consistent with continued food sovereignty, as Dalits pursue new local opportunities for small-scale agriculture (Patel 2009; Van der Ploeg 2014). Becoming a food-sovereign country is important for Nepal because of its geopolitical situation, small-scale agriculture and insignificant share in the international food market. In this sense, land redistribution to those like Dalits, who remain committed to local small-scale agriculture and efforts to improve its productivity, may be a good investment towards increasing domestic supply and creating agricultural self-sufficiency.

However, if we look at the situation in greater depth, things become more complicated. Dalits have not undertaken sharecropping or international migration out of choice, but rather due to the loss of demand for their traditional, artisanal craftwork. Yet even where Dalits show an interest to commit to agriculture, cultural norms of land ownership appear to be blocking that aspiration. Dalits are normally unable to acquire land as higher caste households typically hang on to their land for the social status it brings (Gartaula et al. 2012a). Ironically, this pattern of absentee landlordism is associated with rising land values.^10^ It also contributes to land degradation as farm surpluses are typically not reinvested in agriculture and sharecroppers have little incentive to improve the land they work (Sugden 2013; Adhikari 2011). This is particularly true in situations where the rented land is a secondary source of income for both parties, as is typical for participants in this research. Relations between land owners and renters are in flux at present, and it is unclear which group has the upper hand (Adhikari 2010; Sugden and Gurung 2010; Adhikari and Hobley 2011). It is clear, however, that the current situation is contrary to the goal of food sovereignty for Nepal unless radical land reforms or policies to support sharecroppers were to be undertaken.

Moreover, due to the gradual withdrawal from agriculture of households involved in more lucrative off-farm activities and the adoption of modern lifestyle values, the process of commodification appears to be diminishing the social value of agriculture as a way of life for all social groups. This trend is undermining the moral economy of the family in small-scale subsistence agriculture and is thus an existential threat to small-scale agricultural sustainability and food sovereignty (Amanor 2010). Perhaps for this reason, a Dalit woman in our study (the de facto head of her household) who receives remittances expressed a disinterest in agriculture, just as a higher-caste woman would. She was planning to buy a plot in Pokhara to build a house and stay there in the future, upon the final return of her husband. It is clear from the presented case that any interventions that seek to further Nepalese food sovereignty have to recognize this socio-economic context of migration and urban aspiration. A wholesale cultural reorientation back towards small-scale agriculture or family farming is unrealistic, at least in near future. Nonetheless, poverty and malnutrition continue to be significant concerns in Nepal and attention needs to be directed at how to foster greater care for the land in a new economic environment of interconnection. Are there ways, for example, to encourage remittances to be channelled to constructive investments in small-scale agriculture?

### Food security and wellbeing: women’s agency and their subjective experiences

Differing in their capacity to become food self-sufficient, all the household types appeared to be food secure through various forms of economic access, as described in the previous sections. Given the increased role of women in agriculture and household management, we measured wellbeing indices of these women. When we looked at their wellbeing we observed interesting trends and significant differences, which are determined by the type of household in which they reside. Data presented in Table 4 shows that the wellbeing of women in HHAFs and HHDHs is higher than in other household types, corresponding with their monthly household income. It is also observed that perceived wellbeing is positively correlated with both household income [r(65) = 0.67, *p* < 0.01] and food self-sufficiency [r_s_(65) = 0.46, *p* < 0.01].

**Table 4:** Overall wellbeing of women in the respondent households (N = 59)

Household type	N	Wellbeing index	Monthly income (NPR)
Agriculture and local off-farm			
Informal sector (HHAI)	15	21.9^ac^	8417^ac^
Formal sector (HHAF)	10	27.2^ab^	17,375^ab^
Agriculture and distant off-farm			
Household head (HHDH)	21	24.8^cd^	14,127^c^
Other members (HHOD)	13	22.8^bd^	10,416^b^

^a,b,c^Statistical means with the same letter in the same column are significantly different at 0.05 level, while that of indicated by ^d^ are at 0.1 level (one-way ANOVA)

In terms of income distribution, it is important to note that people in the HHAF category are engaged in formal and salaried jobs where payments are regular and easy to remember, since in most cases they are paid on a monthly basis, with documentary proof. That may be why their income appears to be higher compared to that of other household types. In the HHAI category, where household income comes mainly from agriculture, income is not accounted and wage labour may or may not be remunerated in cash. The cases of migrant households (HHDHs and HHODs), however, are a different matter. Their reported income might not be the total or exact income of the households involved, because it is never clear whether the money sent home by migrant workers is the total or just part of the income earned abroad. In some cases, migrant workers send small amounts required for everyday expenses or for particular household needs, and when they come home they bring more money as a surprise. Therefore, lower income as reported by HHDHs and HHODs is not necessarily because their wage earners make less money but may be due to incomplete reporting.

The wives of migrant workers say that they prefer not to use remittances for daily household expenses but instead keep them for bigger household projects, as explained in the interview transcripts presented below. These women may operate a shop or sell liquor from home to cover daily household costs. Such responsibility increases their self-confidence, self-esteem and feeling of pride that they are also contributing to the household income, as observed in high wellbeing indices in Table 4 for HHDH and HHOD groups. In response to the question “How sure are you that you will be able to get money if you need it for any emergency?”, none of the household respondents from the HHAF, HHDH and HHOD groups that were involved in local and distant high-income off-farm activities answered “not certain”, while about 39 % of the respondents in the HHAI group, whose livelihood is generated from local agriculture and wage labour, gave this answer. In other words, the respondents, the large majority of whom are women, in households that have access to off farm jobs in local formal sector (HHAF) or distant markets (HHDH & HHOD) were more confident. It has to be noted, however, that the confidence of the woman members in these categories largely depended on household structure. Women living with in-laws in joint family settings have less freedom and control over their mobility, and little influence on household decision-making. This diversity of women’s situation in a variety of household settings leads to different outcomes in their wellbeing. Statistically significant results indicate that the wellbeing index of women in the households that were nuclear and headed by women, either due to migration of their husband or for other reasons, was higher (wellbeing index = 25.1) than that of women living in joint or nuclear households headed by men (wellbeing index = 23.1).

Income from non-agricultural employment contributes to household food security in two ways: (1) it can be used to directly purchase food from the market; or (2) it can be used to purchase inputs, improved agricultural technologies and agricultural land, which ultimately increase food availability. In both situations, women’s roles and responsibilities in household decision-making increase in men’s absence (Kelkar 2009; Radel et al. 2012; Gartaula et al. 2010). This is evident from women’s increased agency and stake in household decision-making and their crucial role in household resource management. The following comment by a female household head (HHDH) indicates the impact of male out-migration on female agency:

He has been living abroad for a long time. Since the time we got married, we discuss decisions with each other before taking them. As he is not here, he does not know what is going on in the village. For example, while we were buying this land, he did not know about the price and could not come home just for this purpose. He had to depend on the information I provided. He would not be able to decide without asking me. He said, if everything is okay let’s buy that land.

This evidence of female empowerment contrasts with the following observation about the relative powerlessness felt by a woman living in a joint family setting:

There is no space to speak up for a daughter-in-law. My father-in-law is the one who makes all decisions required for the household. We as household members are supposed to do our job and not concern ourselves with decision-making. I have to ask permission from my mother-in-law even to go to the market and to go to my maita [maternal home]. My parents-in-law decide which crops to grow and where to sell them. My father-in-law manages everything.

Both of these women are food secure in terms of physical and economic access to food. However, the ways they are involved in decision-making and in arrangements for food security differ, as does their resulting level of wellbeing. The woman whose husband does not stay at home and is living in a nuclear family household has to involve herself in activities that were not traditionally women’s work. Women like her appear to be more confident and can exercise more power in household decision-making.

Data also revealed that the quantitative results of the wellbeing index do not seem to be reflected in subjective evaluations of the quality of life women are living. If we look at the size of landholdings (Table 3) along with the number of crops cultivated and animals raised (Table 2), the households engaged in distant off-farm activities have more land under cultivation and more crops cultivated and livestock to take care of. In the absence of their male counterparts, women in these households are more stressed in terms of workload due to their economic responsibilities. It is necessary for some households to use remittances for repaying debts and for food procurement, as their local production is insufficient, but they have a roadmap and dream for a greater future. One of the respondents belonging to the HHDH category said:

Whatever we are doing, it is all for our children; all for our future. We can live in this state, but we don’t want our children to be in this situation. So the money he makes abroad I try not to spend on food and vegetables. I have this business [she prepares liquor for local supply], which is almost enough if I need money, and the grain we produce is enough for almost year round consumption. I use his income only in emergencies; otherwise, I keep it for bigger things.

Apart from their economic role as caretakers of agriculture and local business, women also work as homemakers, which in the absence of their husbands becomes more difficult. These women are unhappy with this situation, but they have to accept the challenge because they do not have other options than to let their husbands go elsewhere to work and send money home. One of the respondents said:

When we hear of accidents abroad, we have very painful time. I always think of him, what he eats, whether he takes care of his health. He has gone there to make money. I am always worried about him getting a good fit for himself there to make money for our and our children’s future. His work is not our will, but an obligation.

This woman’s downcast face and tearful eyes during the interview indicated how difficult it is to live a split family life on the basis of dreams for a better future. As also indicated in the opening interview transcript of this section, it is not women’s preference for their husbands to go abroad. In a similar study carried out in another district, Adhikari and Hobley (2015, p. 17) noted, “Most women indicated they would rather have their husbands at home helping them to bring up the children and to farm the land. However, in all cases, it was not just men wanting to migrate to earn new sources of income, but rather a social expectation in their community that they should migrate”. Embedded in our quantitative data about women’s wellbeing (Table 4) are projections about anticipated future wellbeing. From our series of interactions with those women having higher wellbeing scores, it became evident that they actually down-played their current hardships because they were dreaming about a better future.

We reiterate that the wellbeing of the women left behind is not only a matter of economic gain, but also a social, psychological and relational matter that largely depends on the household type they live in. In all household types, we did not see a significant difference in secure access to food, but the way in which women access food and the way they exercise agency differ. Women in nuclear households have more agency, empowerment and responsibility to run the household in the absence of their husbands. Even though the wellbeing of these women is to a degree influenced by an orientation to the future, they think that their current high levels of labour will pay off in the future for them and their children. In joint households, however, women are part of household labour but have less or no role in household decision-making processes. As they do not have much say within the household, and are possibly not involved in making future plans, this may mute their ambitions for the future. Wellbeing researchers need to be aware of whether people are talking about present or future-oriented perceptions of wellbeing.

Our female respondents told stories about their future plans for the use of remittances, but many indicated that they want to stay away from agriculture, preferring to work in business or trade instead. Some women have already indicated this preference through their involvement in these activities, as in some of the cases presented above. In fact, among the 19 women who participated in in-depth interviews, almost 50 % had bought residential plots either in nearby towns or in Pokhara. In a similar study in the eastern part of Nepal, Gartaula et al. (2012a) also observed a preference to buy residential land over agricultural land. This phenomenon of non-agricultural investment of remittances is also evident in other parts of the world such as China (De Brauw and Rozelle 2008), Mexico (Durand et al. 1996) and Ecuador (Jokisch 2002), and is associated with lower-than-expected levels of agricultural development in the origin areas. This finding not only raises questions about the long-term role of women as caretakers in agriculture, but also indicates a changing perception about food security, where food security through self-production becomes less relevant.

## Conclusions

This study demonstrates the significance of the food wellbeing approach to understanding agrarian development puzzles like that of Nepal’s increasing food security despite the deterioration of its agricultural sector. Combining insights from food security, food sovereignty, and social wellbeing frameworks, we used the food wellbeing approach to illustrate how greater attention to context-specific social relational and subjective factors improves understanding of the drivers of food security in, and sustainability of, small-scale agriculture. Results presented in the paper indicate that additional livelihood choices made by agrarian households, in the form of local and distant off farm employment, have led to changes in labour, gender and caste relations and triggered new economic opportunities as well as responsibilities for traditional low-caste groups and women. The spread of modern lifestyle aspirations has raised consumption demands, while income from small-scale agriculture is unable to meet supply needs for year-round food consumption. The non-agricultural engagement of households, especially in the local formal sector or in the remittance economy, has greatly contributed to increased access, absorption capacity, quality and stability of food supply at the household level.

As landholding size is one of the main limiting factors of small-scale agriculture, our research also looked at land distribution and management. The advent of multiple livelihood activities, especially the out-migration of men, has not only changed how people access food and increase their food security but has also altered land management, as many people who adopt multiple livelihood activities have given their land out on lease or for sharecropping, even though their experience is not always positive. Dalits, who used to be wage labourers and artisans, have now become sharecroppers and migrants. This has given them access to more cultivated land, diversifying their food sources, and increasing their agency. It has also given them access to more non-agricultural income than in the past. For empowerment and food sovereignty, these changes may be seen as good, but in terms of food sovereignty there is also risk as Dalits are largely blocked from acquiring land and, anyway, small-scale agriculture is not their preferred occupation. Further, the growing trend towards short-term sharecropping poses a serious challenge to the sustainability of agricultural ecosystems as neither absentee landlords nor sharecroppers are interested in investing in long-term agricultural practices to support soil fertility, biodiversity and water conservation, or other aspects of ecological sustainability. The current strategy for assuring food security is thus incompatible with food sovereignty.

The main driving force for agrarian change in Nepal is access to non-agricultural income, from within and outside of the country. We observed that people involved in agriculture and local off-farm activities (HHAF) enjoyed the most affluence in terms of income, food security and wellbeing. The changing agrarian landscape has provided opportunities for women’s empowerment by their engagement in the management of households and agriculture and through membership in popular community self-help groups created by NGOs and other development actors. Women’s wellbeing, agency, and empowerment were enhanced in nuclear households with access to remuneration from distant off-farm jobs. However, in the absence of their husbands from home, women bear an added layer of stress, as they shouldered the double responsibility of household management and agricultural work. This burden appears to be a further factor propelling aspirations to move out of agriculture. The wellbeing of women in this category also appears to be significantly influenced by their expectations of this post-agricultural future.

Finally, this paper enlarges the concept of food security by using the food wellbeing approach. This perspective has the advantage of better integrating subjective and social relational considerations into analyses of agrarian change, with the promise of building on food security and food sovereignty approaches. Our results show that even though people in the research area have become food secure in terms of physical and economic access to food, that the social relational status of Dalits has considerably improved, and that people with multiple livelihood activities have better access to food, the way people experience food security is still varied. This is reflected most tellingly in the dilemma faced by many women with out-migrant spouses. While they are now food secure and can aspire to an even better future at some indeterminate time in years to come, food security comes at the cost of the considerable hardship they experience. It is also clear that the current socioeconomic arrangements for women and for Dalits are not conducive to the long-term sustainability of small-scale agriculture in the research area. These findings demonstrate the promise of the food wellbeing approach to broaden the lens of previous food security approaches and to provide more robust measures of the long-term viability of small-scale agriculture in countries like Nepal. Even though the paper does not claim to present a representative picture of Nepal, as it concentrates on a specific case study, it does raise important questions and insights about the balance between food security and wellbeing in the context of the nation-wide changes in agrarian and labour landscapes.

## Footnotes

1 The people’s movement of the 1990s changed Nepal’s political system to a constitutional monarchy that took the country into an open, liberal economy (Hachhethu 2000; Lecomte-Tilouine 2009). In 2006, another political transformation resulted in the country becoming a federal republican state.

2 Out of the country’s total population of 26.5 million, about two million people live outside the country and one in every four households has at least one migrant member (CBS 2011b). Almost 2000 young Nepalese leave the country in search of better wage employment elsewhere every day. The remittances contributed by these migrants account for about 22 % of the national gross domestic product (DOFE 2012). During the period 1994–2012, the country recorded a hundred-fold increase in the migrant population (DOFE 2013). These figures do not include the almost equal number of irregular and unofficial migrants to India and other countries.

3 According to the Nepal Labour Force Survey 2008, about 80 % of women and 87 % of men are economically active. Of those employed, 89 % of women and only 70 % of men are engaged the in agriculture and forestry sector (CBS 2009).

4 The Central Bureau of Statistics has classified farmers across the country based on their landholding size. The classification is different in the *Terai*, Mid-Hill and High-Mountain regions. In the absence of other regional classification, we have adopted this classification representative of Mid-Hills since the site is located in the Mid-Hill region. According to this, households having less than 1.25 acres of land are categorized as small, those with 1.26–4.9 acres as medium, and those with 5.0 or more acres are categorized as large farmers (CBS 2013).

5 Brahmins and Chhetris are the dominant caste/ethnic groups (of Hindu origin) in Nepal in terms of social, cultural, religious and political order. They are also known as higher caste people (Subedi 2011).

6 Dalit is a word coined to denote all untouchable caste groups, such as Kami (blacksmiths), Damai (tailors) and Sarki (cobblers) in Nepal.

7 Janajatis (indigenous nationalities) are generally non-Hindu ethnic groups with distinct identities in terms of their religious beliefs, social practices and cultural values. The National Foundation for Development of Indigenous Nationalities (NFDIN) has identified a total of 59 Janajatis grouped into four categories: 17 in the Mountain Region, 24 in Hills, 7 in the Inner Terai and 11 in the Terai (NFDIN 2009). The Terai is the flat plain region of Nepal’s southern part.

8 Semi-pucca houses have mud floors, concrete walls and clay tile or galvanized iron roofing; pucca houses have concrete floors, walls and roofs; while kachcha houses have mud floors, mud and bamboo walls and thatch roofing.

9 Due to a political misunderstanding, there was an undeclared economic blockade of the border between India and Nepal in late 2015, which did not allow the entry of food and non-food items, including petroleum products, and affected people from all walks of life in Nepal for more than four months. One national newspaper warned, “The school cannot provide snacks to your children due to acute shortage of cooking gas, please do send your children with the required snacks” (The Rising Nepal 2015).

10 According to a recent report, the land price in Nepal has increased by 300 % between 2003 and 2010, mainly due to people’s increased tendency to invest in land and housing during periods of political and economic uncertainty, which further fueled by local banks that had few investment alternatives (UN-HABITAT 2010).
